# The blood-brain barrier and methamphetamine: open sesame?

**DOI:** 10.3389/fnins.2015.00156

**Published:** 2015-05-05

**Authors:** Patric Turowski, Bridget-Ann Kenny

**Affiliations:** Department of Cell Biology, UCL Institute of OphthalmologyLondon, UK

**Keywords:** methamphetamine, blood-brain barrier, endothelial cell, neurovascular unit, pinocytosis, tight junctions, neuroinflammation, CNS chemotherapy

## Abstract

The chemical and electrical microenvironment of neurons within the central nervous system is protected and segregated from the circulation by the vascular blood–brain barrier. This barrier operates on the level of endothelial cells and includes regulatory crosstalk with neighboring pericytes, astrocytes, and neurons. Within this neurovascular unit, the endothelial cells form a formidable, highly regulated barrier through the presence of inter-endothelial tight junctions, the absence of fenestrations, and the almost complete absence of fluid-phase transcytosis. The potent psychostimulant drug methamphetamine transiently opens the vascular blood–brain barrier through either or both the modulation of inter-endothelial junctions and the induction of fluid-phase transcytosis. Direct action of methamphetamine on the vascular endothelium induces acute opening of the blood-brain barrier. In addition, striatal effects of methamphetamine and resultant neuroinflammatory signaling can indirectly lead to chronic dysfunction of the blood-brain barrier. Breakdown of the blood-brain barrier may exacerbate the neuronal damage that occurs during methamphetamine abuse. However, this process also constitutes a rare example of agonist-induced breakdown of the blood-brain barrier and the adjunctive use of methamphetamine may present an opportunity to enhance delivery of chemotherapeutic agents to the underlying neural tissue.

## Properties of the blood brain barrier

The vasculature pervading the nervous system is exceptionally specialized and because of its restrictive nature has been dubbed the blood brain barrier (BBB). The BBB controls the ionic microenvironment for the central nervous system (CNS). It excludes neurotoxic plasma proteins and yet enables cellular and molecular crosstalk between the periphery and the brain parenchyma (Abbott et al., 2010). The importance of the BBB is best illustrated in disease when it is often compromised. Hallmarks of a dysfunctional BBB include unchecked passage of leukocytes, ions, plasma proteins and water, with progression to oedema and irrevocable CNS damage. This contributes to the pathology of classical BBB breakdown such as stroke or trauma (Lo et al., 2003) but also neuroinflammatory diseases such as multiple sclerosis (Alvarez et al., 2011; Argaw et al., 2012). More recently, disorders previously considered exclusively neurological in their etiology, such as Parkinson's and Alzheimer's disease or amyotrophic lateral sclerosis, have also been found to be accompanied or even caused by significant BBB dysfunction (Drozdzik et al., 2003; Jeynes and Provias, 2011). From a pharmacological point of view, the BBB presents a formidable impediment to efficient delivery of therapeutic agents into the brain. Therefore, much BBB research focuses on finding ways to breach the barrier for therapeutic purposes (Patel et al., 2009).

## Cells contributing to the BBB

The properties of the BBB are defined by the neurovascular unit, which is the functional association of cerebrovascular endothelial cells (ECs) with neurons and non-neuronal cells including pericytes, astrocytes, perivascular macrophages, and microglia (Daneman, 2012). The actual barrier operates mostly at the level of the microvascular EC, which display a sophisticated tight junction (TJ) network, a lack of fenestrae and low levels of fluid phase transcytosis (pinocytosis), all of which restricts the passage of molecules and cells. The luminal face of the BBB ECs is covered by a glycocalyx, a non-selective first line filtration barrier (Van Teeffelen et al., 2007).

On the abluminal side cerebral ECs interact with surrounding pericytes with which they share a basement membrane. Pericyte coverage at the BBB and blood-retinal barrier is the highest of all vascular beds, with EC to pericyte ratios typically ranging between 1:1 and 3:1 (Armulik et al., 2011). Pericytes are essential for BBB formation during development and also strongly influence its mature properties and function during health and disease. For instance, pericytes suppress EC functions that normally facilitate transcellular permeability and leukocyte infiltration in the periphery (Armulik et al., 2010; Daneman et al., 2010). Dysfunctional pericyte-endothelial interactions appear to drive the pathogenesis of neurodegenerative diseases such as Alzheimer's disease (Montagne et al., 2015).

Astrocytes ensheath CNS blood vessels and, either directly or via the parenchymal basement membrane, regulate neuronal function and coordinate multitudinous signals from neurons, the BBB and their microenvironment during such instances as neurovascular coupling linking neural activity to blood flow (Petzold and Murthy, 2011). Astrocytes also strongly influence both BBB TJs and transport properties (Abbott et al., 2010).

CNS immune cells include microglia, which are highly ramified, phagocytic cells found throughout the CNS. They contribute to innate and adaptive immune responses and neuronal homeostasis (Daneman, 2012). Perivascular BBB macrophages reside between the basal lamina and astrocytic foot processes, where they are involved in first line CNS immune surveillance and antigen presentation (Daneman, 2012). Their activation affects the BBB with an increased secretion of cytokines contributing to increased permeability and leukocyte infiltration (Denieffe et al., 2013).

## Molecular characteristics of the vascular barrier of the CNS

TJs are a dynamic protein complex connecting adjoining epithelial and ECs (Steed et al., 2010). In addition, by separating luminal from abluminal cellular surfaces, TJs, and to a lesser degree adherens junctions (Dejana and Orsenigo, 2013), are responsible for regulating the passage of solutes and cells through the paracellular space, for the establishment and maintenance of apico-basal polarity and for cell-cell-contact mediated intracellular signaling. TJs are composed of transmembrane proteins which regulate cell-cell interactions, the lateral organization of junction strands as well as associations with cytoplasmic TJ proteins. Claudins (CLDNs) are by far the most dominant transmembrane protein found in BBB TJs. They constitute a large family of proteins, which form the structural fabric of TJs through homophilic and heterophilic, *cis* and *trans* interactions. Depending on the isoform, claudins can be pore or fence forming. At least three CLDNs (3, 5, and 12) have been identified in BBB ECs. CLDN5 contributes to BBB function in that it regulates paracellular transport of small (<800 dalton) blood solutes (Nitta et al., 2003). Occludin is another transmembrane protein present in all epithelial and endothelial TJs, which regulates certain aspects of paracellular diffusion and TJ organization *in vitro* (Steed et al., 2010). Other transmembrane proteins of BBB TJs include junctional adhesion proteins and tricellulin. Cytoplasmic TJ proteins, such as zona occludens proteins ZO-1, ZO-2, and ZO-3, cingulin, binding partitioning defective proteins PAR-3 and PAR-6, MAGI and MUPP1 and AF-6, densely pack the space beneath the cell membrane and dynamically link the integral membrane proteins to the cytoskeleton and a multitude of intracellular signaling proteins. However, many of the described molecular features of TJs are not exclusive to the BBB and cannot fully account for the exceptional solute and electrical impermeability of the cerebral vasculature.

The healthy BBB endothelium lacks fenestrae and exhibits very low levels of transcytosis (Daneman, 2012). This might be due to the lack of a glycoprotein called plasmalemmal vesicle associated protein-1 (PLVAP or PV-1). PV-1 was first identified as a caveolar protein and thus with a role in vesicular trafficking. Subsequently it was identified as a key element in the formation of fenestrae diaphragms. At the healthy BBB, pericytes suppress PV-1's expression, which conversely is enhanced during pathological BBB breakdown (Shue et al., 2008; Daneman, 2012). A pericyte-deficient BBB features increased macromolecular permeability with increased cytoplasmic vesicles but with intact polarization and a continued lack of fenestrae (Armulik et al., 2010; Daneman et al., 2010).

In addition to restricting unwanted passage of cells and solutes, ECs have to allow regulated nutrient and immune cell entry into the CNS and ensure removal of toxic substances and waste. This is achieved by five different mechanisms: passive diffusion, ATP-binding cassette (ABC) transporters, solute carriers, transcytosis, and transendothelial migration of leukocytes (Saunders et al., 2013). BBB ECs display exceptional apico-basal polarization with respect to the expression profile of many of these transport systems, enabling preferential transport to or from brain. Lipid soluble, non-polar molecules can diffuse across the BBB unrestricted, as do oxygen and carbon dioxide, moving along concentration gradients. ABC transporters, such as P-glycoprotein and multidrug resistance-associated proteins, function as active efflux pumps and transport lipid soluble compounds out of the CNS. In turn, many of the essential polar nutrients, such as glucose and amino acids, are transported into the CNS by specific solute carriers. Larger molecular weight proteins and peptides generally enter the CNS via endothelial transcytosis. Whilst leukocytes appear to migrate through both the healthy and diseased vascular BBB, diapedesis appears to be primarily transcellular at the intact BBB (Engelhardt and Wolburg, 2004; Daneman, 2012).

## Modulation of the BBB under pathophysiological conditions

On the one hand a pathologically weakened BBB could provide a better immune response, but on the other hand have profound adverse effects on the CNS, inciting neuronal damage and degeneration. A disrupted BBB is characterized by aberrations in both paracellular and transcellular pathways (Stamatovic et al., 2008). Faulty paracellular transport develops following changes in the phosphorylation and adhesive properties of TJ and adherens junction complexes, resulting in altered junctional protein interactions, localization, or even down-regulation. Concomitant reorganization of the actin cytoskeleton also contributes to permeability, with contractile forces thought to render the paracellular space more compliant to modification (Stamatovic et al., 2008). Matrix metalloproteinases (MMPs), most notably MMP9, are activated by reactive oxygen species, vascular endothelial growth factor and inflammatory cytokines in many CNS pathologies (Daneman, 2012). MMP activation leads to the degradation of EC basement membrane and enhanced phosphorylation and cleavage of TJ proteins, with subsequent degradation of the interendothelial junctions. The development of faulty transcellular transport has been less extensively studied, however early brain oedema, and increased expression of caveolin-1, has been found to precede disruptions to TJ protein expression (Nag et al., 2007). Potential mediators of this early event in BBB dysfunction include vascular endothelial growth factor, which induces pinocytic vesicles in blood-retinal barrier (Hofman et al., 2000) and blood tumor barrier endothelium in conjunction with increased expression of caveolin-1 and −2 (Zhao et al., 2011). Bradykinin also induces transcellular transport within blood tumor barrier endothelium with increased expression of caveolin-1 and −2 (Liu et al., 2010).

## Methamphetamine-induced BBB dysfunction

Methamphetamine (METH) is a highly addictive CNS stimulant with demonstrated neurotoxicity. Long-term damage to monoaminergic nerve terminals is caused by excitotoxicity, mitochondrial dysfunction, and increased production of reactive oxygen and nitrogen species (Quinton and Yamamoto, 2006). METH readily crosses the BBB due to its small size and lipophilicity. It is also a substrate for the organic cation transporters OCT3 and OCTN2 (Wu et al., 1998a,b), with the latter undisputedly expressed in BBB ECs (Friedrich et al., 2003). METH rapidly accumulates in the brain parenchyma of rodents (Martins et al., 2011), and this suggests that, unlike other lipophilic drugs, METH evades BBB efflux pump activity. Moreover, METH exposure alters the permeability of the BBB and this is likely to exacerbate its neurotoxicity.

In reviewing data relating to METH-induced BBB opening we would like to begin with our observation that such meta-analysis is complicated due to the highly varied protocols used to mimic METH intoxication, both *in vitro* and *in vivo*. Most studies do not report detailed kinetic or concentration dependent methods. Furthermore, literature on METH-induced BBB breakdown is replete with studies reporting the effects of METH at concentrations that are hugely in excess of those found in typical drug abuse, and are in fact associated with lethality (Takayasu et al., 1995). Very high concentrations of METH in preclinical experiments in rodents unsurprisingly lead to neurotoxic stress, hyperthermia and death, and it is likely that BBB breakdown in these instances is a consequence of METH-induced brain death. In this review we focus on studies that have employed METH concentrations that are likely to reflect relevant (but not lethal) abuse scenarios.

A number of studies have shown significant breakdown of the BBB in rodents in response to METH administration (Table 1). However, few of the studies controlled the plasma concentrations of METH during the observation period.

**Table 1:** **Studies investigating the effect of METH on BBB integrity**.

	**Effective METH dosage causing barrier breakdown**	**Time scale of observed changes**	**Experimental model**	**References**
*In vivo* studies	9 mg/kg	>1 h	Rat	Kiyatkin et al., 2007
	40 mg/kg or daily 10 mg/kg	1.5 h to 3 days	Mouse	Bowyer et al., 2008
	Repeated dose of 1.5–10 mg/kg	9 days	Mouse	Ramirez et al., 2009
	9 mg/kg	>1 h	Rat	Sharma and Kiyatkin, 2009
	4 × 10 mg/kg	3 days	Mouse	Kuroda et al., 2010
	3 mg/kg or 9 mg/kg (acute); 2–3 mg/kg self-adminstered (chronic)	24 h (acute); 10 days (chronic)	Rat	Kousik et al., 2011
	30 mg/kg	24 h	Mouse	Martins et al., 2011
	3 × 4 mg/kg over 9 h	3–24 h	Mouse	ElAli et al., 2012
	10 mg/kg	1 h	Mouse	Park et al., 2013
	3 × 4 mg/kg over 9 h	10 h	Mouse	Urrutia et al., 2013
*In vitro* studies	10–50 nM	24 h	Astrocyte-EC co-culture (human)	Mahajan et al., 2008
	50 μM	2 h	Primary EC (human)	Ramirez et al., 2009
	>10 μM	≥1 h	hCMEC/D3 cell line (human)	Park et al., 2012
	1 μM	<1 h	primary EC (rat)	Martins et al., 2013
	10 μM	1 h	hCMEC/D3 cell line (human)	Park et al., 2013

**Figure 1:**
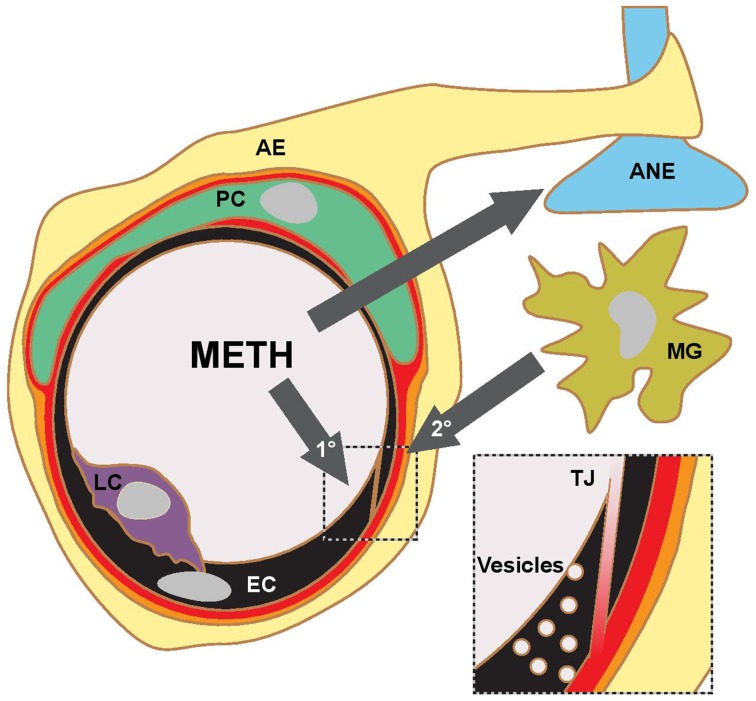
**METH action in the CNS and proposed pathways of BBB opening**. Current literature supports a model, where METH affects the transport properties of BBB ECs directly (1°) and indirectly (2°) through inflammatory signaling following glial activation, aminergic nerve (ANE) damage and hyperthermia. The indirect pathway is likely to involve microglia (MG) activation and transmigrating leukocytes (LC). Current experimental evidence suggests that the opening of the BBB occurs on the level of TJs and fluid-phase vesicular transport. AE, astrocyte endfoot; PC, pericyte.

Martins et al. (2011) find that a single 30 mg/kg METH i.p. injection in mice leads to a peak plasma concentration of ca. 30 μM after about 1 h. In human subjects typical METH abuse leads to plasma concentrations in the mid μM range with concentrations above 30 μM considered lethal (Cook et al., 1992; Melega et al., 2007). Acute administration of METH at such concentrations in rodents consistently leads to BBB breakdown, visualized by measuring plasma protein (albumin or IgG) accumulation in the parenchyma. Significant accumulation of these proteins was generally observed after several hours (Table 1). Protocols mimicking chronic abuse fail to demonstrate significant BBB dysfunctions but transient BBB opening may have been missed due to the experimental paradigm used (Kousik et al., 2011). METH induces some BBB breakdown across the entire CNS but studies have noted a certain tropism, with protein extravasation significantly more prominent in the hippocampus (Bowyer et al., 2008; Sharma and Kiyatkin, 2009; Martins et al., 2011). These data are in line with others indicating that the BBB of the hippocampus is particularly sensitive (Terrando et al., 2014; Montagne et al., 2015). *In vitro* studies using primary or immortalized monocultures of brain microvascular ECs also found enhanced permeability in the presence of METH, albeit many studies employed concentrations of METH greater than 50 μM (Mahajan et al., 2008; Ramirez et al., 2009; Martins et al., 2013). The simplest paradigm arising from *in vivo* and *in vitro* data suggests that METH induces endothelial permeability, leading to a transient opening of the BBB, which then contributes to the neurotoxic features of METH (see Figure 1). However, this simple model should be treated with caution. Careful comparison of the *in vitro* and *in vivo* datasets indicates that barrier dysfunction occurs with significantly different kinetics following METH exposure. *In vitro*, using endothelial monocultures, METH induces permeability within less than 1 h. In contrast, *in vivo*, METH effects on the BBB generally take more than an hour to develop and are often observed to persist chronically for many hours/days. This could be due to a lack of sensitivity in detecting leakage at the BBB through extravasated plasma proteins. More direct and acute measurements of BBB breakdown could be employed to exclude this possibility (Hudson et al., 2014). Nevertheless, the reported kinetic discrepancies indicate that METH affects BBB integrity in at least two ways (Figure 1). Firstly, it acts acutely and directly on brain microvascular ECs, inducing permeability within minutes. Here the effective concentration range of METH is relatively narrow (Martins et al., 2013). Secondly, METH induces BBB breakdown in a delayed and possibly chronic, inflammatory fashion, as a consequence of neuronal damage, glia activation and hyperthermia (O'Shea et al., 2014). Enhanced BBB sensitivity to METH of FosB null mice supports the existence of this second, indirect pathway (Kuroda et al., 2010).

The strong effects of low micromolar, or even submicromolar, concentrations of METH in monocultures of brain microvascular ECs point toward the existence of a direct binding protein for METH (Mahajan et al., 2008; Ramirez et al., 2009; Martins et al., 2013). METH can bind and affect the activity of a wide variety of proteins, most of which are expressed in neurons and nerve terminals^1^. METH stimulation of the trace amine-associated receptor 1, a G-protein coupled receptor, is considered of particular importance (Bunzow et al., 2001). It is yet unclear whether this receptor, or any of the other relevant targets of METH, is expressed in brain microvascular ECs but preliminary data suggests that this might be the case^2^.

The responses of brain microvascular ECs to METH are varied. Reduction of TJ proteins, in particular of occludin but also CLDN5, has been observed in rodents (Martins et al., 2011) and in cultures of brain microvascular ECs (Ramirez et al., 2009; Park et al., 2012). Importantly, the effect on the junctions usually requires high concentrations of METH and takes hours to become noticeable. TJ protein downregulation is likely to be a consequence of oxidative stress, with antioxidants relieving some symptoms of METH-induced BBB dysfunction (Park et al., 2012, 2013; Urrutia et al., 2013). ARP2/3-mediated endocytosis also appears to play a role (Park et al., 2013). Increased MMP activity in response to METH contributes to BBB dysfunction and TJ downregulation, with significant attenuation observed in the presence of MMP inhibitors (Martins et al., 2011; Urrutia et al., 2013). With neuroinflammation and associated cytokine release being another hallmark of METH intoxication (Goncalves et al., 2008; O'Shea et al., 2014), it is likely that MMPs are derived by glial cells (Conant et al., 2004) or invading leukocytes, just as in many other instances of pathological BBB breakdown (Daneman, 2012).

Using a primary EC model highly predictive of the intact BBB (Hudson et al., 2014), our group has found that moderate concentrations of METH, corresponding to plasma concentrations in typical drug abusers, lead to rapid endothelial leakage through a fluid phase, i.e., vesicular pathway, with TJs left intact (Martins et al., 2013). This data is in agreement with a paradigm of enhanced vesicular transport preceding TJ breakdown at the BBB in certain pathophysiological conditions (see above). METH-induced fluid phase transcytosis is dependent on endothelial nitric oxide synthase activity but other key mechanistic questions, e.g., pertaining to the potential regulation by PV-1 or by adjacent pericytes (and other components of the neurovascular unit which might regulate vesicular transport at the BBB) remain. It also needs to be established if both para- and transcellular BBB permeability are induced by METH and if so under which conditions, especially since, as seen with leukocyte migration, the route may depend on the respective tightness of the experimental model used (Ransohoff and Engelhardt, 2012).

## Exploiting METH for delivering therapeutics to the CNS

The observation that BBB breakdown is transient and thus resembles an agonist-driven (or therapeutic) opening of the BBB, suggests that METH and related compounds could be used to enhance the transport of other drugs to the CNS. Indeed, this is an idea proposed as early as 2007 (Kast, 2007) and, given that METH is FDA approved, has been picked up repeatedly since (Focosi and Kast, 2010; Sardi, 2011; Capeloa et al., 2014). However, preclinical or clinical data in support of such a therapeutic strategy have not yet been reported. Before METH is trialed as an adjuvant to CNS chemotherapy, a number of aspects of METH-induced pathology should be considered. For instance, is there a need to harness the neurostimulatory or neurotoxic action of METH? How does METH affect diseased CNS cells, e.g., glioblastoma (Capeloa et al., 2014)? Will the apparent tropism of METH restrict its effectiveness to parts of the CNS such as the hippocampus?

## Concluding remarks

Research into the effects of METH on the BBB should be considered topical and highly relevant, and not just with regard to understanding METH abuse and its neurological consequences. METH affects barrier function of BBB ECs directly but also induces a response, which is reminiscent of neuroinflammation, in that it involves glial activation, cytokine, and MMP release as well as oxidative stress. Mechanistic insights into METH action are likely to enhance our understanding of the BBB, particularly the molecular and cellular mechanisms of its pathophysiological regulation, as well as reveal vital insight into the specificity of BBB efflux pumps for monoamines. Finally, research into METH may even facilitate our reach toward disease targets currently hidden behind the BBB.

### Conflict of interest statement

The authors declare that the research was conducted in the absence of any commercial or financial relationships that could be construed as a potential conflict of interest.

## Footnotes

^1^http://www.drugbank.ca/drugs/DB01577#targets

^2^http://www.proteinatlas.org/ENSG00000146399-TAAR1/tissue/cerebral+cortex

## Abbreviations

BBB: blood-brain barrier
CNS: central nervous system
EC: endothelial cell
TJ: tight junction
CLDN: claudin
PV-1: plasmalemmal vesicle associated protein-1
ABC: ATP-binding cassette (transporters)
MMP: matrix metalloproteinases
METH: methamphetamine.
